# A geospatial analysis of liver transplant centers and alcohol-related liver disease across the United States

**DOI:** 10.1016/j.liver.2025.100290

**Published:** 2025-07-18

**Authors:** Luke M. Tomasovic, Jeremy R. Ellis, Alexander C. Schulick, Parth Agrawal, Anmol Warman, Andrew M. Cameron, Elizabeth A. King

**Affiliations:** a Johns Hopkins University School of Medicine, Baltimore, MD, USA; b Medical Scientist Training Program, Johns Hopkins University School of Medicine, Baltimore, MD, USA; c Division of Transplantation, Department of Surgery, Johns Hopkins University School of Medicine, Baltimore, MD, USA

**Keywords:** Liver transplantation, Geographic disparities, Alcohol-related liver disease, Alcohol use disorder

## Abstract

Alcohol-related liver disease (ARLD) represents a major cause of end-stage liver disease and has surged as a leading indication for liver transplantation. This study investigates geographic disparities in liver transplant center availability relative to the regional burdens of ARLD mortality and alcohol use disorder (AUD) prevalence in the U.S. Using state-level data from publicly available databases, we evaluated the relationships between liver transplant center density, ARLD mortality, and AUD prevalence. We also developed two novel metrics: the AUD prevalence-to-transplant recipients (AUDT) ratio and the ARLD deaths-to-transplant recipients (ARLDT) ratio. These ratios served as proxies for assessing disparities between the need for and access to liver transplant services. Our findings reveal that while AUD prevalence and AUDT ratios did not significantly vary with transplant center density, higher ARLD mortality per capita and ARLDT ratios were correlated with lower transplant center density. States without a transplant center also experienced significantly higher ARLD mortality per capita compared to states with at least one transplant center per 100,000 square miles. These findings underscore the significant role of geographic factors in accessing transplant care and suggest that barriers to transplant centers may contribute to outcome disparities among patients with ARLD. The study also highlights the need for targeted healthcare planning and policy interventions to enhance liver transplant access, particularly in regions with disproportionately high ARLD burdens and limited transplant infrastructure. Future research should utilize more granular geographies, such as transplant referral regions, and incorporate covariates related to overall healthcare infrastructure and access.

## Introduction

Alcohol-related liver disease (ARLD) is one of the leading causes of end-stage liver disease and can present as severe alcoholic hepatitis, chronic liver disease, or acute on chronic decompensation [1]. The number of patients with ARLD who were listed for liver transplant increased by 63 % from 2007 to 2017 [2]. In 2022, approximately 40.8 % of liver transplant cases in the U.S. were performed for ARLD, up from 17 % in 2012 [3].

ARLD is closely linked to alcohol use disorder (AUD), a condition characterized by problematic patterns of alcohol use and an inability to limit consumption despite experiencing clinically significant impairment or distress [4]. As of 2022, AUD affects approximately 29.5 million people over the age of 12 in the U.S., a 103 % increase from 14.5 million in 2019 [5,6]. Numerous studies have highlighted the significant impact of the COVID-19 pandemic on rising alcohol consumption across the U. S., which likely contributed to the growing prevalence of AUD during this time [7,8]. Among individuals with AUD, 10–15 % will develop liver cirrhosis [9], and in 2022 alone, alcohol was involved in 46 % of liver disease deaths [10].

With the rise of both AUD and ARLD, coupled with a persistent organ shortage in the U.S., ensuring equitable access and allocation of donor livers has become more critical than ever. Before receiving a liver transplant, a patient must be referred to a transplant center for evaluation and, if warranted, placed on the transplant waitlist. Yet, the number and location of liver transplant centers vary widely across states, influenced by factors such as healthcare infrastructure, economic resources, and regional policies [11].

Several studies have established that regional variations in the number of liver transplant centers can manifest as disparities in transplant waitlist access and outcomes [12,13]. Across various fields of organ transplantation, including lung, kidney, and liver, greater travel distance from a transplant center is linked to lower transplant rates and higher risks of allograft failure [14-18]. In the case of liver transplant specifically, an analysis of liver transplant candidates in Oklahoma and Texas found that mortality risk was higher in patients living over 30 miles from a transplant center [16]. In another study, patients eligible for liver transplant who lived further from a transplant center had a reduced likelihood of waitlist registration and liver transplant, along with a decreased 5-year survival [17].

The objective of this study is to quantitatively assess the geographic distribution of AUD prevalence, ARLD mortality, and liver transplant centers across U.S. states and census divisions. To do so, we generated two empirical measures. The AUD prevalence-to-transplant recipients (AUDT) ratio compares the number of individuals with AUD to the number of ARLD-related liver transplant recipients. The ARLD deaths- to-transplant recipients (ARLDT) ratio compares the number of ARLD deaths to the number of ARLD-related liver transplant recipients. Higher AUDT or ARLDT values in certain regions may indicate disparities between the need for and access to transplant care.

## Methods

### Liver transplant center and recipient data collection

We used liver transplant center data from the Organ Procurement & Transplantation Network (OPTN) Membership Directory [19]. The search was restricted to hospitals with an active OPTN member status. Since the study focused on liver transplants related to alcohol consumption, children’s hospitals were excluded. To provide a consistent frame of reference for comparisons of liver transplant availability between states and census divisions, we divided the number of transplant centers in each region by its respective land area. The resulting transplant center density was then normalized by 100,000 square miles, which is approximately the average size of a U.S. state. Transplant center density was also calculated relative to state population.

State-level data on liver transplants and waitlist additions for alcohol-related conditions were obtained from the OPTN Advanced Reports dashboard. Data were collected for the 2022 calendar year. ARLD-related indications for liver transplant included the following: alcoholic cirrhosis, alcoholic cirrhosis with hepatitis C, acute alcoholic hepatitis, acute alcohol-associated hepatitis with or without cirrhosis, alcohol-associated cirrhosis without acute alcohol-associated hepatitis. For the indications defined above, there were no liver transplant recipients under the age of 18 in 2022.

### Geographic measurements and definitions

To estimate the number of liver transplant centers per square mile within each census division, state area measurements were derived from the 2010 Census Bureau’s Master Address File/Topologically Integrated Geographic Encoding and Referencing (MAF/TIGER^®^) database [20]. Census divisions are geographic groupings of states used for statistical analysis. The nine U.S. census divisions include: New England, Middle Atlantic, East North Central, West North Central, South Atlantic, East South Central, West South Central, Mountain, and Pacific.

### ARLD mortality and AUD prevalence data collection

We obtained state-level data on ARLD mortality from the CDC WONDER online databases [21]. Underlying cause of death statistics in 2022 were obtained for the following ICD-10 codes: K70.0 (Alcoholic fatty liver), K70.1 (Alcoholic hepatitis), K70.2 (Alcoholic fibrosis and sclerosis of liver), K70.3 (Alcoholic cirrhosis of liver), K70.4 (Alcoholic hepatic failure), and K70.9 (Alcoholic liver disease, unspecified). The inclusion of ICD-10 codes K70.0-K70.9 is consistent with previous studies estimating ARLD prevalence and mortality [22,23]. State-level AUD data were sourced from the SAMHSA 2021–2022 National Survey on Drug Use and Health (NSDUH) [24]. The NSDUH is an annual survey using multistage area probability sampling of the U.S. population. While this enables state-level estimation, NSDUH estimates rely on self-report, are subject to recall and social desirability bias, and are not clinically verified. These factors may result in underreporting, particularly in states with limited health access or higher stigma surrounding alcohol use. We obtained estimates of the resident population aged 18 years and older in each state from the U.S. Census Bureau [25].

### Choropleth map generation

To construct the choropleth maps, we utilized shapefiles from the U. S. Census Bureau’s TIGER/Line files, which provide geographic and cartographic boundary data for various geospatial analyses. The shapefiles were imported into R, where they were processed and filtered using the sf (simple features) package. We merged these shapefiles with state-level data of the AUDT and ARLDT ratios. AUDT and ARLDT ratios were mapped to each state by joining the shapefiles with the corresponding values using the dplyr package for data manipulation. For each state, the values were represented in a color gradient, using ggplot2 for visualization. To include Alaska and Hawaii in the choropleth maps, their positions were manually adjusted using the coord_sf() function to recenter the maps around the Pacific region to prevent distortions in the geographic layout. The final choropleth maps excluded Puerto Rico and other U.S. territories.

### Statistical methods and analysis

**Fig. 1.**
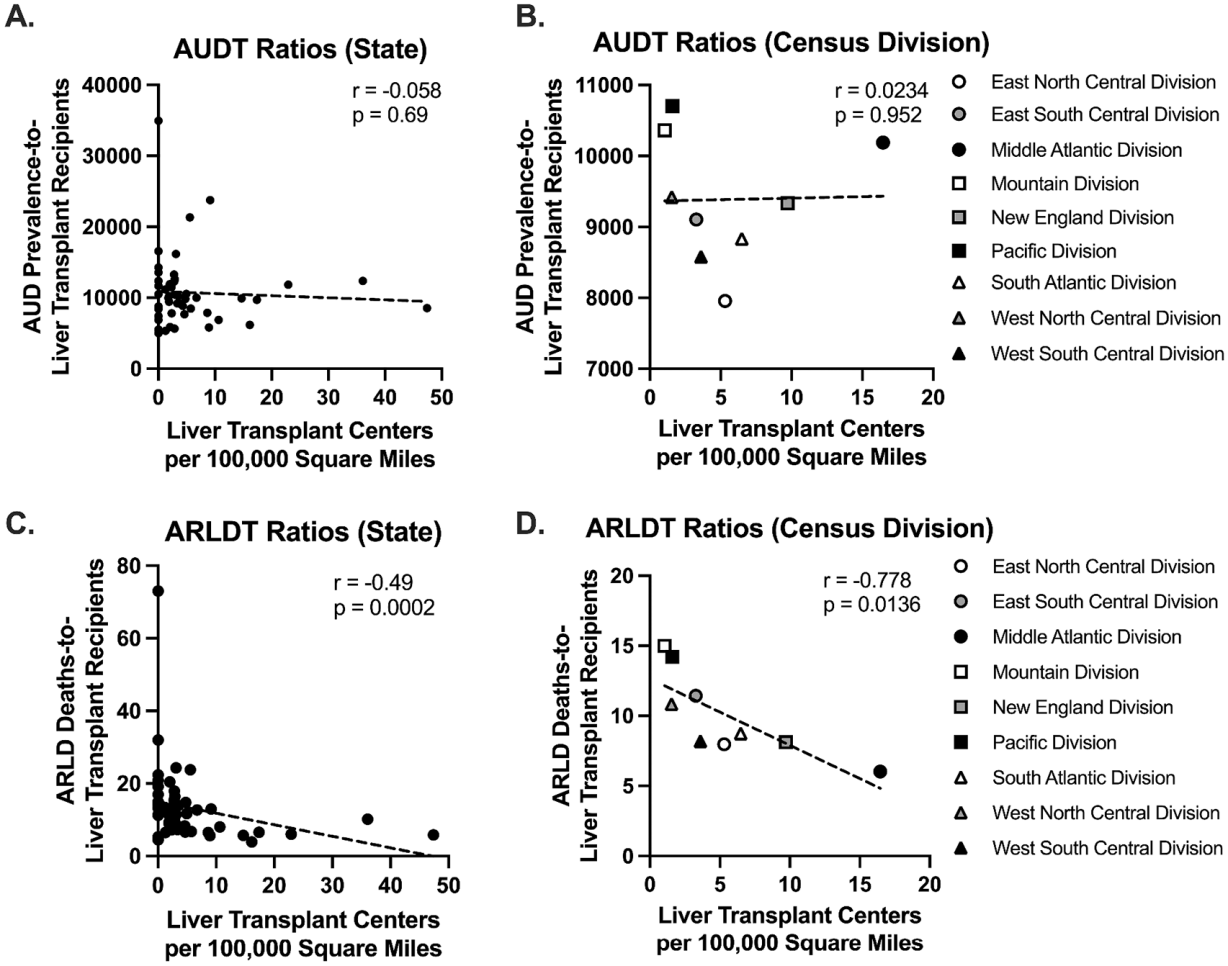
Correlations between liver transplant center density and AUD prevalence-to-transplant recipients (AUDT) or ARLD deaths-to-transplant recipients (ARLDT) ratios across U.S. states and census divisions. Scatter plots of (A,B) AUDT and (C,D) ARLDT ratios versus liver transplant centers per 100,000 square miles across states and census divisions, respectively. Simple linear regressions are represented by dashed, black lines. Nonparametric Spearman correlation coefficients (r) are shown for Fig. 1A and B, and Pearson correlation coefficients (r) are provided for Fig. 1C and D. P-values were calculated from two-tailed *t*-tests.

For the datasets in each figure, D’Agostino and Pearson omnibus normality tests were performed in GraphPad Prism (RRID:SCR_002798) to assess whether the data followed a Gaussian distribution. The results subsequently informed the selection of appropriate statistical tests. Data in Figs. 1D, 4C, and Supplementary Figure 1 were assumed to be sampled from Gaussian distributions. Data in Figs. 1A-C, 3A-B, 4A-B and Supplementary Figs. 2-3 were sampled from non-Gaussian distributions. For XY plots, Pearson correlation coefficients were calculated for data with Gaussian distributions, while nonparametric Spearman correlation coefficients were used for data with non-Gaussian distributions. For bar graphs, ordinary one-way ANOVA followed by post hoc Tukey’s multiple comparisons test was used to assess statistical significance between datasets with Gaussian distributions, and the Kruskal-Wallis test followed by post hoc Dunn’s multiple comparisons test was applied for non-Gaussian datasets.

## Results

The number of liver transplant centers, recipients of liver transplants for alcohol-related indications, AUD prevalence per capita, ARLD deaths per capita, and AUDT and ARLDT ratios are presented in Table 1. Notably, the five states with the highest ARLD mortality rates per capita—South Dakota, New Mexico, Wyoming, Alaska, and Montana—do not have a single liver transplant center within their borders. In 2022, ARLD deaths per 100,000 residents in these states were notably higher than the national average of 11.9, with rates of 32.4 in South Dakota, 30.3 in New Mexico, 28.4 in Wyoming, 26.2 in Alaska, and 23.8 in Montana. A similar pattern emerges when examining AUD prevalence. Among the 11 states with the highest AUD prevalence per capita, six states—North Dakota, Rhode Island, Wyoming, Alaska, Nevada, and Vermont—lack a liver transplant center.

Correlations between liver transplant center density and the AUDT and ARLDT ratios are shown in Fig. 1. At the state-level, there was no significant correlation between the number of liver transplant centers per 100,000 square miles and the AUDT ratios (Fig. 1A) (*r* = −0.058, *p* = 0.69). However, the number of transplant centers per area was significantly negatively correlated with the ARLDT ratios across states (*r* = −0.49, *p* = 0.0002) (Fig. 1C), indicating that a lower transplant center density is associated with higher ARLDT ratios. Evaluating the ARLDT ratios with respect transplant to center density across census divisions also revealed a significant negative correlation (*r* = −0.778, *p* = 0.0136) (Fig. 1D). The same negative trend was not observed between liver transplant center density and the AUDT ratio across census divisions (*r* =0.0234, *p* = 0.952) (Fig. 1B).

**Fig. 2.**
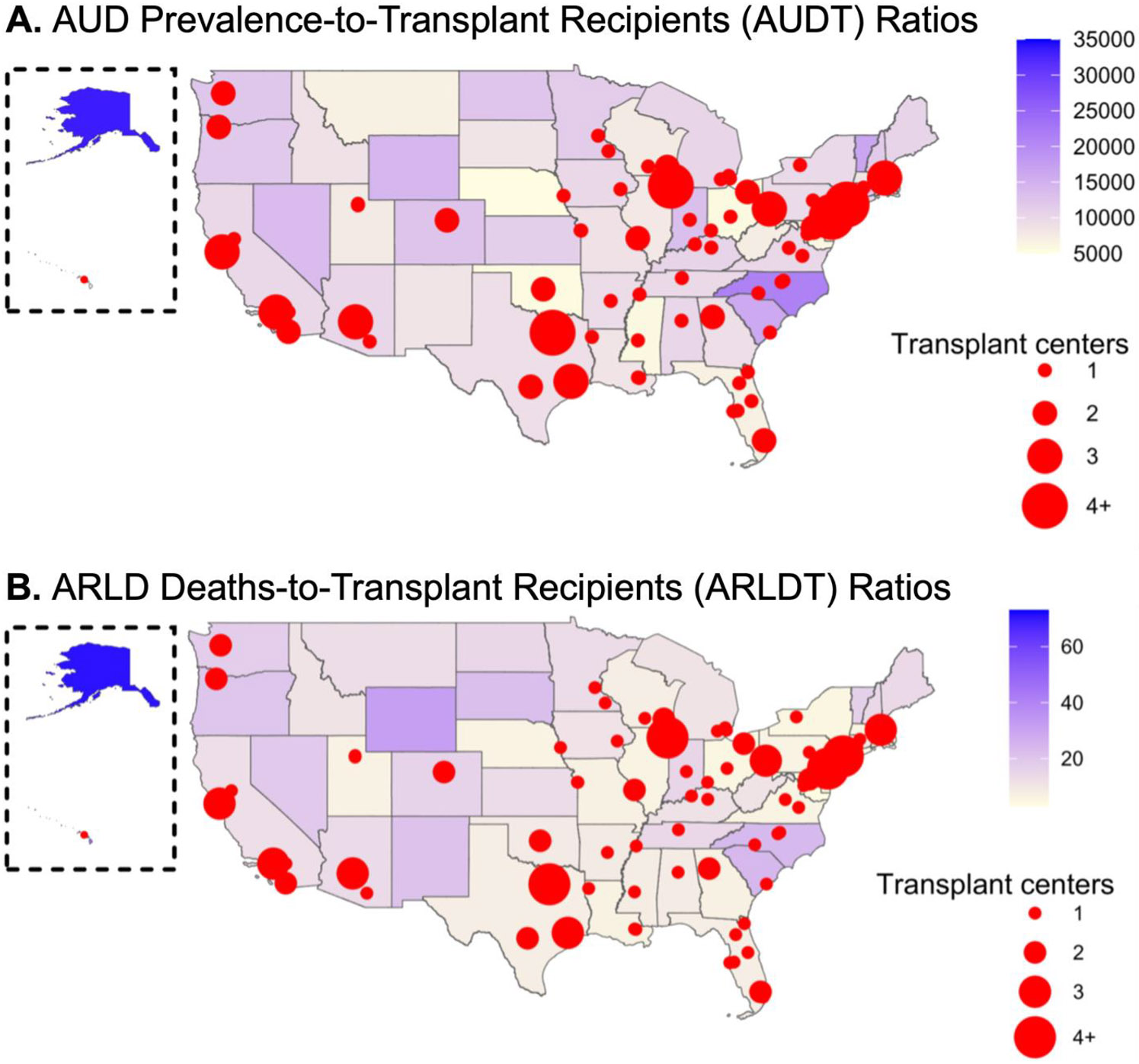
Geographic distribution of liver transplant centers in relation to state-level AUD prevalence-to-transplant recipients (AUDT) or ARLD deaths-to-transplant recipients (ARLDT) ratios. State-level choropleth map illustrating the (A) AUDT and (B) ARLDT ratios across the U.S. Higher values are represented by darker shading. Red circles indicate cities containing at least one liver transplant center, with circle size representing intracity transplant center quantity.

Choropleth maps containing state-level AUDT and ARLDT ratios were subsequently constructed to visualize these ratios in relation to the locations of liver transplant centers across the U.S. These maps highlight regional differences in state-level AUDT ratios (Fig. 2A) and ARLDT ratios (Fig. 2B). Each map also shows that regions with a higher density of liver transplant centers, such as the Northeast, have lower AUDT and ARLDT ratios. Meanwhile, these ratios are typically higher in areas with fewer centers.

**Fig. 3.**
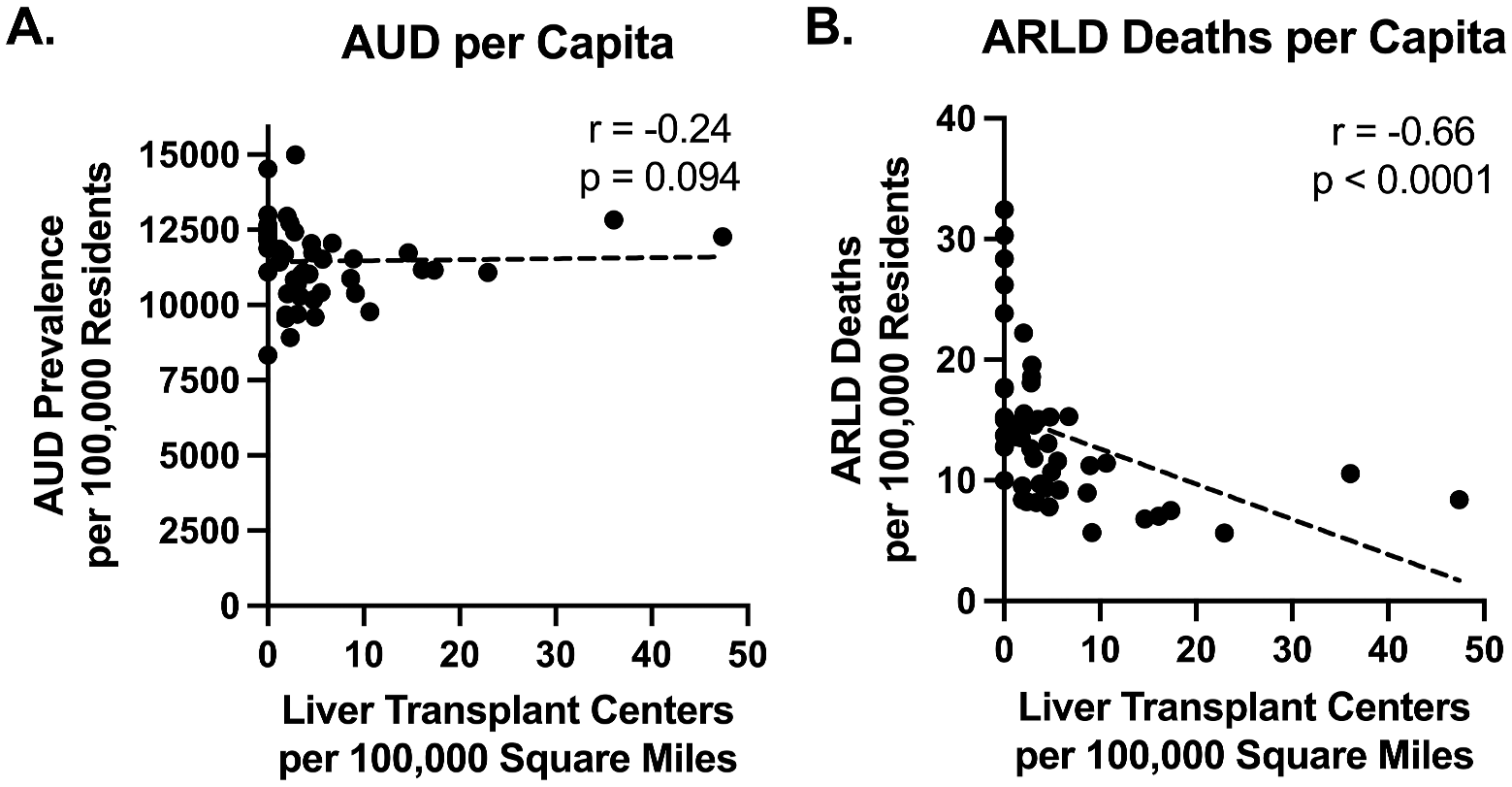
State-level correlations between liver transplant center density and AUD prevalence or ARLD deaths per capita. Scatter plots of the number of liver transplant centers within each state versus (A) AUD prevalence per 100,000 residents and (B) ARLD deaths per 100,000 residents. Simple linear regressions are represented by dashed, black lines. Nonparametric Spearman correlation coefficients (r) for each dataset are shown, and p-values were calculated from two-tailed *t*-tests.

To gain a clearer understanding of the factors driving differences in AUDT or ARLDT ratios between states, AUD prevalence per capita and ARLD deaths per capita were plotted against the number of liver transplant centers per area. Transplant center density was not significantly associated with AUD prevalence per capita (Fig. 3A) (r = −0.24, p = 0.094). The relationship between ARLD-related liver transplant waitlist additions and the number of liver transplant centers per area was also insignificant (r = −0.082, p = 0.57) (Supplementary Fig. 2A. In contrast, ARLD deaths per capita was significantly inversely correlated with transplant center density (Fig. 3B) (r = −0.66, p < 0.0001). The relationships between these variables were similar when analyzed at the level of census divisions, in which ARLD deaths per capita (Supplementary Fig. 1B) was significantly negatively associated with the number of liver transplant centers per 100,000 square miles (r = 0.84, p = 0.0043).

Transplant center densities per 1 million state residents were also calculated to provide an alternative metric for transplant center availability. There was no significant correlation between the number of liver transplant centers per 1 million residents and the AUDT ratios (Supplementary Fig. 3A) (*r* = −0.13, *p* = 0.36) or the AUD prevalence per capita (Supplementary Fig. 3C) (*r* = −0.13, *p* = 0.36). Meanwhile the number of transplant centers per 1 million residents was significantly negatively correlated with state-level ARLD ratios (Supplementary Fig. 3B)) (*r* = −0.38, *p* = 0.006) and ARLD deaths per capita (Supplementary Fig. 3D) (*r* = −0.41, *p* = 0.003).

**Fig. 4.**
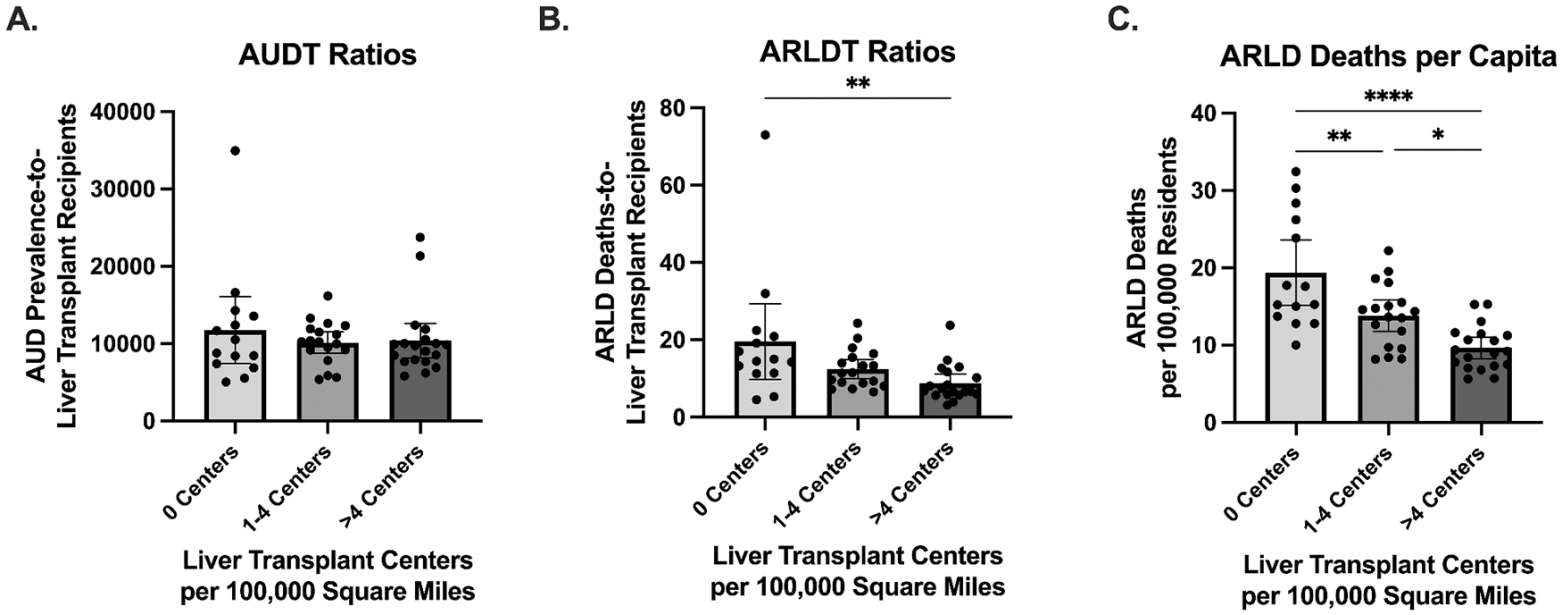
Grouped comparisons of ARLD deaths-to-transplant recipients (ARLDT) ratios, AUD prevalence-to-transplant recipients (AUDT) ratios, and ARLD deaths per capita. Bar graphs showing the (A) AUDT ratios, (B) ARLDT ratios, and (C) ARLD deaths per 100,000 residents across groups. States are grouped by their respective number of transplant centers per 100,000 square miles. Error bars represent the 95 % confidence interval. Statistical significance determined by Kruskal-Wallis test with post-hoc Dunn’s multiple comparisons test (A,B) or ordinary one-way ANOVA followed by post hoc Tukey’s multiple comparisons test (C) (*p < 0.05, **p < 0.01, ***p < 0.001).

To further explore the relationships between liver transplant center density, the AUDT or ARLDT ratios, and ARLD deaths per capita, states were categorized into 3 groups: states with 0 centers per 100,000 square miles, states with 1–4 centers per 100,000 square miles, and states with greater than 4 centers per 100,000 square miles. These categories were selected to capture the broad range of liver transplant center accessibility, from limited to extensive transplant infrastructure. The AUDT ratios (Fig. 4A) were not significantly different between states with 0, 1–4, and greater than 4 centers per area. In contrast, ARLDT ratios (Fig. 4B) were significantly higher among states without a transplant center compared to those with >4 centers per area (*p* = 0.0031). Similarly, states without a transplant center had significantly higher ARLD deaths per capita (Fig. 4C) compared to states with 1–4 centers per area (*p* = 0.0064) and states with >4 centers per area (*p* < 0.0001).

## Discussion

In this national study examining the geographic distribution of liver transplant centers, AUD prevalence, and ARLD mortality, we found that residents in states without a transplant center received liver transplants for ARLD at rates comparable to those in states with transplant centers, despite experiencing a significantly higher ARLD mortality burden per capita. Additionally, regions with lower liver transplant center density had significantly higher ratios of ARLD deaths relative to ARLD-related liver transplant recipients, suggesting that access to liver transplant care may not be adequate in regions with a disproportionately higher ARLD burden. While these findings may be attributable to geographic disparities in liver transplant center access, they also reflect broader challenges in healthcare access, including the multiple steps involved in transplant referral, evaluation, and listing. States with more transplant centers likely have better overall hepatology care infrastructure, contributing to earlier diagnosis and management of ARLD, independent of transplant listing. Thus, the observed associations between transplant center density and ARLD outcomes may partially reflect systemic disparities in liver disease care rather than transplant access alone.

In contrast to our findings related to ARLD mortality, our analysis of AUD prevalence per capita and AUDT ratios did not reveal statistically significant relationships with transplant center availability. These results suggest that while the prevalence of AUD is widespread, it is not disproportionately higher in states with limited access to liver transplant care. This lack of correlation may also be explained by the fact that only a small proportion of individuals with alcohol use disorder progress to end-stage liver disease and ultimately require transplantation. Moreover, the relationships in Fig. 3 suggest that the ARLDT ratio may be a more useful metric for gauging differences in ARLD mortality rates across regions, rather than reflecting differences in liver transplant rates.

The AUDT and ARLDT metrics reflect different stages of disease progression and seek to quantify imbalances between the need for and access to liver transplantation. AUDT primarily captures upstream gaps in diagnosis and treatment of AUD, while ARLDT better represents latestage inequities in liver transplant access and outcomes. This distinction may explain why only ARLDT showed a significant association with transplant center density. However, it is important to note that plotting ratios such as ARLDT or AUDT against transplant center density could amplify observed effects because the denominator of these ratios is closely tied to transplant center density. This mathematical coupling may result in a stronger apparent association than is truly present. To mitigate this concern and provide a more direct understanding of disparities, we highlight our findings on absolute ARLD death rates by transplant center density (Figs. 3 and 4C), which show that regions with lower center density experience significantly higher ARLD mortality per capita.

We also recognize that transplant center density measured per 100,000 square miles may be confounded by state size, as large, sparsely populated states may appear underserved by this metric alone. Our supplementary analysis using population-normalized center density confirmed that the observed associations are not solely attributable to state area.

The overarching results of this analysis align with previous research, which has shown that geographic proximity to transplant centers is a key determinant of access to liver transplant and contributes to mortality risk [11,12,16,17,26,27]. Based on CDC data, New York has the lowest age-adjusted death rates from liver disease, with the entire population residing within 150 miles of at least one liver transplant center. In contrast, New Mexico and Wyoming, two states with over 95 % of their populations living >150 miles from a liver transplant center, have the highest age-adjusted death rates [28]. These findings are consistent with our data, as both New Mexico and Wyoming are located in the Mountain census division, a region characterized by the lowest density of liver transplant centers and the highest ARLD mortality rates and ARLDT ratios.

A recent study suggests that the relationship between liver-related mortality and liver transplant rates varies several-fold between states, with the lowest rates of liver transplant in states with the most liver-related deaths [29]. This study extends these findings by focusing specifically on ARLD, a growing public health issue due to the rising prevalence of alcohol consumption and AUD in the U.S. The significant differences in ARLDT ratios across regions with varying levels of transplant center availability reinforce the utility of this metric in identifying regions that may benefit from increased access to liver transplant services.

Several limitations should be considered when interpreting the findings of this study. The AUDT and ARLDT ratios are presented as unadjusted correlations with the density of liver transplant centers, limiting the conclusions that can be drawn regarding causality. Key factors such as socioeconomic status and insurance coverage may confound the relationships observed between transplant center density and ARLD outcomes. Our analysis also does not account for the multiple barriers encountered at different steps before transplant listing and surgery, including patient referral and specialist evaluation, which may cumulatively contribute to regional differences in liver transplant rates.

Our analysis also does not control for differences in waitlist processes that may exist between individual transplant centers. In future studies, considering variations in factors such as MELD score, average time on the waitlist, patient age, and organ availability at the center level could improve generalizability.

The analysis also relies heavily on state-level data, which may obscure intrastate disparities, particularly in larger states where healthcare access can vary significantly across rural and urban areas. Future studies could benefit from incorporating more granular data, such as county-level information, to better capture these intrastate variations. Similarly, transplant referral patterns frequently cross state boundaries; therefore, analyses based on referral flows or proximity to the nearest transplant center would more accurately capture disparities in access.

The study focused solely on data from 2022, which may not fully capture trends that have developed over time or account for delayed impacts from the COVID-19 pandemic on healthcare access and liver transplant services. The impact of transplant access on ARLD mortality would likely manifest over a longer time frame; thus, future studies should adopt a longitudinal or time-lagged approach to strengthen causal interpretations. Given limited state-level data on ARLD prevalence, the study uses ARLD mortality as a proxy for ARLD prevalence. However, ARLD prevalence and mortality may not be correlated to the same extent in different regions, and the proportion of ARLD cases requiring transplant may not be homogeneous across regions. Future studies should compare ARLD prevalence across states while incorporating biomarkers to assess disease severity, with the aim of identifying patient populations with ARLD who might benefit from increased liver transplant rates.

We did not perform formal modeling of the effects of adding transplant centers to underserved regions and their impact on adjacent areas. Such analyses would be informative, and we recommend this as an area for future work, potentially leveraging referral region and patient flow data.

In states with limited healthcare infrastructure, the addition of a transplant center alone may not be sufficient to overcome barriers such as poverty, low health literacy, and long travel distances. Low-cost interventions based on telemedicine and mobile health technologies, similar to those described by Stotts et al. [30] could be leveraged to address gaps in ARLD management. Focusing on early diagnosis, addiction treatment, and access to general hepatology care may offer greater benefit in these regions than increased transplant infrastructure alone.

## Conclusion

In conclusion, this study sheds light on the geographic disparities in access to liver transplant for high-risk patients across the U.S. We show that regions with lower transplant center density exhibited significantly higher ARLD deaths per capita and higher ratios of ARLD deaths to liver transplant recipients. These findings provide a useful metric for pinpointing regions with disproportionate ARLD mortality and potentially inadequate transplant services or infrastructure to support referral to the nearest transplant center. These findings underscore the multifactorial nature of transplant care, suggesting that improving not just the availability of transplant centers but also access to existing primary prevention resources could improve outcomes for liver transplant candidates with ARLD. Efforts to address these disparities may involve a combination of optimizing referral networks, creating satellite clinics for transplant evaluation in underserved areas, and providing financial and logistical support for patients requiring long-distance travel to transplant centers. Ultimately, such strategies would be beneficial steps towards enhancing equity in liver transplantation and reducing the burden of ARLD mortality across the U.S.

## Supplementary Material

1

Supplementary material associated with this article can be found, in the online version, at doi:10.1016/j.liver.2025.100290.

## Figures and Tables

**Table 1:** Regional Distribution of Liver Transplant Centers, Liver Transplant Recipients, AUD Prevalence, and ARLD Mortality Across the United States. Summary of the number of liver transplant centers, liver transplant recipients, AUD prevalence, ARLD mortality, and population data for different regions across the U.S. AUD prevalence data represent estimates and 95 % confidence intervals. Median and interquartile ranges are calculated from state-level values.*Represents the number of liver transplant recipients for the following indications: alcoholic cirrhosis, alcoholic cirrhosis with hepatitis C, acute alcoholic hepatitis, acute alcohol-associated hepatitis with or without cirrhosis, and alcohol-associated cirrhosis without acute alcohol-associated hepatitis.

Region	Number of Liver Transplant Centers	Number of Liver Transplant Recipients*	AUD Prevalence per 100,000	ARLD Deaths per 100,000	AUD Prevalence to Liver Transplant Recipient Ratio	ARLD Deaths to Liver Transplant Recipient Ratio	Population (Aged 18 Years and Older)
Median (Interquartile Range)	2 (0 - 3)	37 (22 - 71)	11,537 (10,778 - 12,323)	12.8 (9.3 - 15.3)	9895 (7848 - 11,879)	11 (7 - 15)	3507,735 (1449,432 - 5954,700)
*United States*	115	3168	11,280 [9855 - 12,891]	11.9	9288	9.8	260,836,730
*East North Central Division*	16	517	11,150 [9797 - 12,667]	11.2	7956	8.0	36,889,851
Illinois	5	136	10,880 [9521 - 12,406]	9.0	7890	6.5	9861,901
Indiana	1	43	10,843 [9313 - 12,590]	12.6	13,272	15.5	5263,114
Michigan	3	82	10,721 [9571 - 11,991]	11.8	10,361	11.4	7924,418
Ohio	4	183	11,537 [10,284 - 12,921]	11.2	5796	5.6	9193,508
Wisconsin	3	73	12,038 [10,347 - 13,962]	13.1	7663	8.3	4646,910
*East South Central Division*	6	166	9913 [8394 - 11,671]	12.5	9104	11.4	15,245,667
Alabama	1	37	9564 [8111 - 11,244]	8.4	10,243	9.0	3962,734
Kentucky	2	32	9606 [8174 - 11,259]	10.7	10,530	11.7	3507,735
Mississippi	1	40	10,376 [8793 - 12,206]	15.5	5868	8.8	2261,996
Tennessee	2	57	10,169 [8574 - 12,21]	15.3	9836	14.8	5513,202
*Middle Atlantic Division*	18	373	11,413 [10,028 - 12,964]	6.8	10,190	6.0	33,302,996
New Jersey	2	68	11,081 [9494 - 12,896]	5.6	11,843	6.0	7267,590
New York	8	186	11,732 [10,404 - 13,204]	6.8	9895	5.7	15,687,863
Pennsylvania	8	119	11,164 [9834 - 12,647]	7.5	9707	6.5	10,347,543
*Mountain Division*	9	232	12,142 [10,328 - 14,224]	17.6	10,363	15.0	19,801,565
Arizona	4	62	11,013 [9254 - 13,059]	15.0	10,250	14.0	5770,187
Colorado	3	55	14,993 [12,960 - 17,282]	19.5	12,606	16.4	4624,351
Idaho	0	20	11,879 [10,9 - 14,044]	15.2	8765	11.3	1475,629
Montana	0	16	12,381 [10,383 - 14,699]	23.8	6880	13.3	889,114
Nevada	0	23	12,535 [10,683 - 14,655]	17.7	13,559	19.2	2487,994
New Mexico	0	24	12,243 [10,453 - 14,291]	30.3	8437	20.9	1653,831
Utah	2	28	8924 [7562 - 10,502]	8.2	7806	7.2	2449,192
Wyoming	0	4	12,663 [10,622 - 15,30]	28.4	14,286	32.0	451,267
*New England Division*	7	162	12,351 [10,488 - 14,492]	10.8	9333	8.1	12,241,593
Connecticut	2	30	12,830 [10,830 - 15,136]	10.6	12,381	10.2	2895,175
Maine	0	12	11,095 [9269 - 13,229]	15.0	10,517	14.3	1137,442
Massachusetts	5	81	12,265 [10,500 - 14,280]	8.4	8547	5.9	5644,540
New Hampshire	0	12	12,254 [10,401 - 14,383]	15.2	11,665	14.5	1142,307
Rhode Island	0	23	12,998 [10,882 - 15,454]	13.7	5029	5.3	889,822
Vermont	0	4	12,462 [10,637 - 14,550]	12.8	16,584	17.0	532,307
*Pacific Division*	16	474	12,144 [10,875 - 13,546]	16.1	10,701	14.2	41,765,607
Alaska	0	2	12,549 [10,641 - 14,742]	26.2	34,953	73.0	557,060
California	11	368	12,054 [10,967 - 13,234]	15.3	9998	12.7	30,523,315
Hawaii	1	5	10,392 [8696 - 12,375]	5.7	23,755	13.0	1142,870
Oregon	2	37	12,954 [11,137 - 15,16]	22.2	11,914	20.4	3403,149
Washington	2	62	12,432 [10,701 - 14,397]	18.1	12,310	17.9	6139,213
*South Atlantic Division*	19	629	10,416 [9081 - 11,926]	10.3	8832	8.7	53,335,689
Delaware	0	18	12,256 [10,450 - 14,325]	10.0	5517	4.5	810,269
District of Columbia	2	13	18,200 [15,613 - 21,108]	7.3	7663	3.1	547,328
Florida	7	255	9780 [8697 - 10,983]	11.4	6884	8.0	17,948,469
Georgia	2	94	10,292 [8958 - 11,799]	8.2	9200	7.3	8402,753
Maryland	2	87	11,178 [9590 - 12,991]	7.0	6190	3.9	4818,071
North Carolina	3	41	10,413 [8925 - 12,116]	11.6	21,345	23.8	8404,094
South Carolina	1	25	9697 [8130 - 11,527]	14.6	16,154	24.3	4164,762
Virginia	2	80	11,737 [10,458 - 13,150]	7.8	10,001	6.7	6816,709
West Virginia	0	16	8336 [6757 - 10,244]	12.8	7415	11.4	1423,234
*West North Central Division*	8	211	11,875 [9998 - 14,050]	13.7	9420	10.8	16,736,808
Iowa	1	29	11,702 [9844 - 13,856]	13.5	9991	11.6	2476,028
Kansas	1	23	11,432 [9711 - 13,413]	13.6	11,165	13.3	2246,318
Minnesota	2	49	12,725 [10,746 - 15,8]	14.7	11,486	13.3	4423,022
Missouri	3	60	11,027 [9192 - 13,175]	9.4	8845	7.5	4813,049
Nebraska	1	33	11,857 [10,098 - 13,875]	14.4	5358	6.5	1491,246
North Dakota	0	7	14,528 [12,234 - 17,169]	17.6	12,380	15.0	596,486
South Dakota	0	10	12,154 [10,171 - 14,462]	32.4	8394	22.4	690,659
*West South Central Division*	16	404	10,998 [9784 - 12,352]	10.5	8580	8.2	31,516,954
Arkansas	1	24	9664 [8097 - 11,497]	9.5	9457	9.3	2348,518
Louisiana	3	48	11,526 [9810 - 13,497]	9.2	8473	6.8	3528,548
Oklahoma	2	59	10,834 [9233 - 12,673]	18.6	5631	9.7	3066,654
Texas	10	273	11,077 [10,030 - 12,218]	9.7	9159	8.0	22,573,234

## Abbreviations:

ARLD: Alcohol-related liver disease
ARLDT: Alcohol-related liver disease deaths-to-transplant recipients
AUD: Alcohol use disorder
AUDT: Alcohol use disorder prevalence-to-transplant recipients
CDC: Centers for Disease Control and Prevention
MELD: Model for end-stage liver disease
NSDUH: National survey on drug use and health
OPTN: Organ Procurement & Transplantation Network
SAMHSA: Substance Abuse and Mental Health Services Administration
