# Supplementary material for: A geospatial analysis of liver transplant centers and alcohol-related liver disease across the United States
Source: J Liver Transpl. Author manuscript; Available in PMC 2025 Aug 27. (PMC12380387; doi:10.1016/j.liver.2025.100290)
Supplement: 1 [file NIHMS2103938-supplement-1.docx]

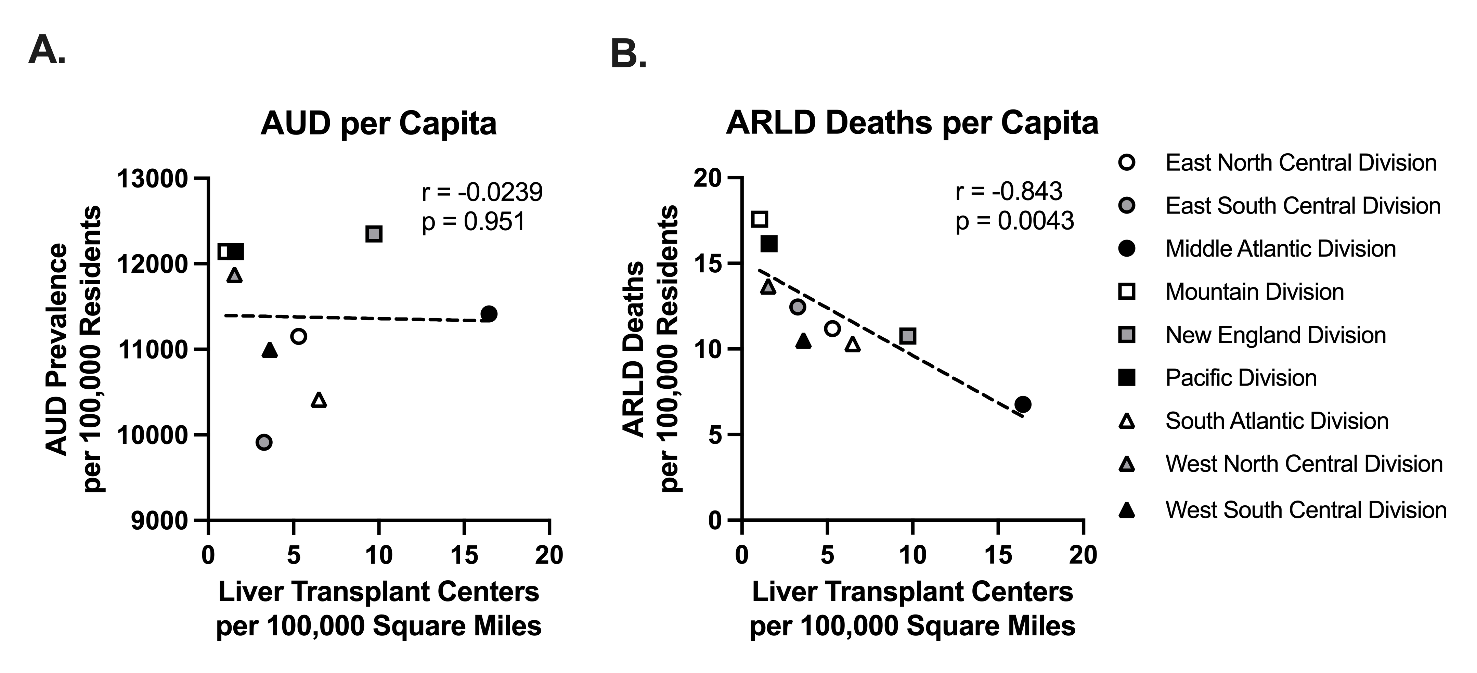


**Supplementary Figure 1. Census-Level Correlations Between Liver Transplant Center Density and AUD Prevalence or ARLD Deaths per Capita**

Scatter plots of the number of liver transplant centers per 100,000 square miles versus **(A)** AUD prevalence per 100,000 residents and **(B)** ARLD deaths per 100,000 residents. Simple linear regressions are represented by dashed, black lines. Pearson correlation coefficients (r) for each dataset are shown, and p-values are calculated from two-tailed t-tests.


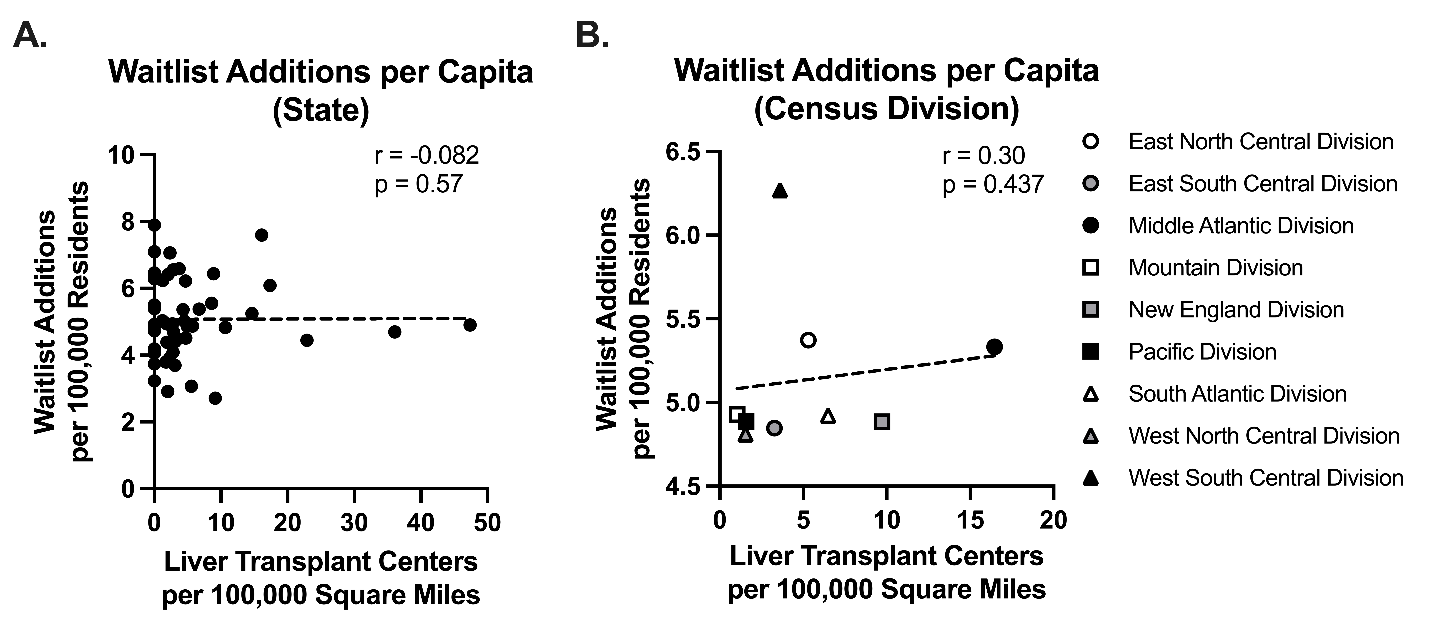


**Supplementary Figure 2. Correlations Between ARLD-Related Liver Transplant Waitlist Additions and Transplant Center Density Across U.S. States and Census Divisions**

Scatter plots of ARLD-related waitlist additions per 100,000 residents versus the number of liver transplant centers per 100,000 square miles across each **(A)** state and **(B)** U.S. census division. Simple linear regressions are represented by dashed, black lines. Pearson and nonparametric Spearman correlation coefficients (r) are shown for Figures 1a and 1b, respectively. P-values are calculated from two-tailed t-tests.

**Supplementary Figure 3. State-Level Correlations Between Liver Transplant Center Density per Population and AUD Prevalence-to-Transplant Recipients (AUDT), ARLD Deaths-to-Transplant Recipients (ARLDT) Ratios, AUD prevalence per capita, and ARLD deaths per capita**

Scatter plots of the number of liver transplant centers per 1 million residents versus **(A)** AUDT ratios, **(B)** ARLDT ratios, **(C)** AUD prevalence per 100,000 residents and **(D)** ARLD deaths per 100,000 residents. Simple linear regressions are represented by dashed, black lines. Nonparametric Spearman correlation coefficients (r) for each dataset are shown, and p-values were calculated from two-tailed t-tests.
